# Cytological Grading of Breast Carcinomas and Its Prognostic Implications

**DOI:** 10.7759/cureus.29385

**Published:** 2022-09-20

**Authors:** Koshalya Rajendran, Muthu Sudalaimuthu, Shivashekar Ganapathy

**Affiliations:** 1 Pathology, SRM Medical College Hospital and Research Centre, Chengalpattu, IND

**Keywords:** breast cancer, cytological grading, core needle biopsy, fine needle aspiration cytology, histological grading, breast carcinoma

## Abstract

Introduction

Determining the histological grade of breast carcinomas before mastectomy is necessary to decide about neoadjuvant chemotherapy. Core needle biopsies used for this purpose often under-grade the tumour. The grade obtained from fine needle aspiration cytology samples will help in such situations and whenever biopsy is not done, as in a resource-poor setup. Many studies are being done to find out the cytological grading system that correlates well with histological grading.

Methods

This study was done between 2016 and 2019 including the cases in which both modified radical mastectomy and fine needle aspiration of the tumour had been done. Robinson’s cytological grading was done in Papanicolaou and haematoxylin & eosin (H&E) stained cytology smears and correlated with modified Bloom-Richardson histologic grading done in modified radical mastectomy specimens. We also studied the prognostic significance of Robinson’s method by studying the association between cytological grade and lymph node metastasis.

Results

Sixty cases were studied. The two methods had the same grade in 49 (81.7%) cases. They showed a significant positive correlation (Spearman correlation coefficient 0.848, p-0.0001), significant association (Chi-square test, p-0.0001), and substantial agreement (kappa value 0.72). Multiple regression analysis showed chromatin score and nucleoli score as the most influential parameters. Lymph node metastasis showed significant association with cytological grade (p-0.0003), cell dissociation score (p-0.0001), nucleoli score (p-0.01), and chromatin score (p-0.04).

Conclusion

Robinson’s cytological grading is a simple, reliable adjunct/alternative to core needle biopsies for grading breast carcinomas before mastectomy. Hence, it can be made a part of routine cytology reporting of breast carcinomas. Further long-term studies will help in confirming its prognostic significance.

## Introduction

According to the GLOBOCAN 2020 statistics, breast cancer is the most common cancer among females as well as the leading cause of cancer-related mortality in them [1]. The prognostic significance of histological grading in invasive breast carcinomas is well established and is routinely done in almost all centres [2,3]. In breast carcinomas, histological grading done on excised tumour specimens is usually taken as the gold standard [4,5]. However, tumour grade in excision specimens may change or become difficult to assess following neoadjuvant chemotherapy. In such circumstances, it becomes essential to know the actual pre-chemotherapy grade of the breast carcinoma by other means to determine the prognosis of the patient [6,7]. Similarly, it may be necessary to know the grade of breast carcinoma before tumour excision to decide about neoadjuvant therapy [4,5,8]. In these circumstances, the grade of breast carcinoma can be obtained only from fine needle aspiration cytology (FNAC) smears or core needle biopsy (CNB) specimens done before mastectomy. Grading obtained from CNB specimens, although widely used, has shown a concordance of only 59-75% with the final histological grade in previous studies [4,9,10]. Hence, there is a need for a cytological grading system that can complement CNB grading. Many methods have been suggested for the cytological grading of breast carcinomas [11,12]. Recent studies have shown that the grading method suggested by Robinson et al strongly correlated with the histological grade [13-16].

The aims of this study were to grade invasive breast carcinomas in FNAC smears by Robinson’s method and correlate it with the final histological grade obtained from tumour excision specimens. We also studied the possible prognostic significance of Robinson’s method by studying its association with lymph node metastasis.

## Materials and methods

This study was conducted between 2016 and 2019 in SRM Medical College Hospital and Research Centre, a tertiary care health institution in Chengalpattu, Tamil Nadu, India. Institutional Review Board approval was obtained from the Institutional Scientific and Ethical Committee of SRM Medical College Hospital and Research Centre (approval number: 1024/IEC/2016). Cases of invasive breast carcinoma in which both the modified radical mastectomy (MRM) and FNAC of the breast lump had been done in our institution were included in our study. All these MRM specimens were received in the histopathology lab within six hours after excision. Informed consent was obtained from the patients before including them. Samples which were received after chemotherapy were excluded from our study. Cases in which the FNAC had inadequate material with less than six clusters were also excluded from our study. In all these patients, fine needle aspiration (FNA) was performed using a 20ml disposable syringe and 22-gauge needle using the aspiration technique with multi-directional passes. FNA smears were stained with Papanicolaou and hematoxylin & eosin (H&E) staining. In Robinson’s cytological grading system, six different cytological parameters, namely cell dissociation, cell size, cell uniformity, nucleolus, nuclear margin and nuclear chromatin were used to grade the tumour (Table 1).

**Table 1:** Robinson’s method for cytological grading

Parameter	Score 1	Score 2	Score 3
Cell dissociation	Cells mostly in clusters	Mixture of single cells and clusters	Mostly single cells
Cell size	1-2 times size of red blood cells	3-4 times size of red blood cells	≥ 5 times size of red blood cells
Cell uniformity	Monomorphic	Mildly pleomorphic	Pleomorphic
Nucleoli	Indistinct	Noticeable	Prominent
Nuclear margins	Smooth	Slightly irregular/folds and grooves	Buds and clefts
Chromatin	Vesicular	Granular	Clumped and cleared

Histological grading was done by Nottingham’s modified Bloom-Richardson method on the H&E stained sections of the tumour in the mastectomy specimen. Tubule formation, nuclear pleomorphism, and mitotic count were used to do histological grading (Table 2). Slides were examined using Olympus CX21 microscope (Olympus Corporation, Shinjuku City, Tokyo, Japan) with field view number 18 and field diameter 0.45 mm for the high power view. Sections from the lymph nodes obtained from MRM specimens were assessed to determine lymph node metastasis.

**Table 2:** Nottingham’s modified Bloom-Richardson histological grading *Mitotic count assessed according to the field diameter (0.45mm) of the microscope we used.

Parameter	Score 1	Score 2	Score 3
Tubule formation	>75% of the tumour	10 to 75% of the tumour	<10% of the tumour
Nuclear pleomorphism	Mild. Small, regular and uniform cells	Moderate with visible nucleoli	Marked with prominent nucleoli
Mitotic count per 10 High power field^*^	0 – 5	6 – 10	>10

Statistical analysis was done using IBM SPSS Statistics for Windows, Version 22.0 (Released 2013; Armonk, New York, United States). Association between the two grading systems, between cytological grade and lymph node metastasis, and between various cytological parameters and lymph node metastasis were assessed using Chi-square test. Kappa value of agreement was used to measure the strength of agreement between the two grading systems. Correlation between the two grading systems was assessed by Spearman's rank correlation coefficient. Multiple linear regression analysis was done to find out the most influential parameters for cytological grading. A p-value of less than 0.05 was considered statistically significant.

## Results

**Figure 1:**
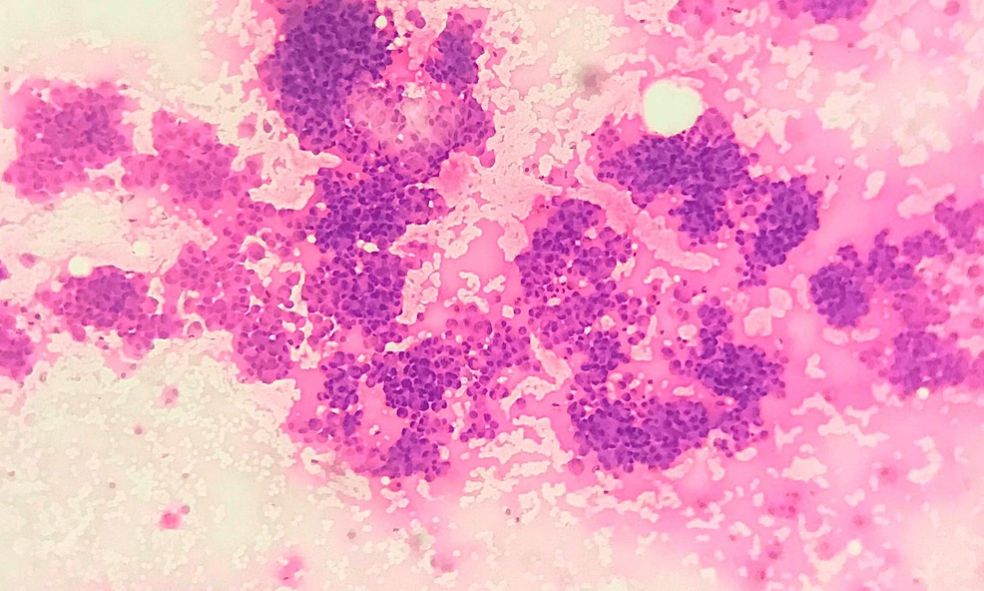
Grade I tumour – cell arrangement FNAC smear showing high cellularity and cells arranged predominantly in clusters – Cell dissociation score 1 (FNAC - H&E x 40) FNAC: fine needle aspiration cytology; H&E: hematoxylin and eosin

**Figure 2:**
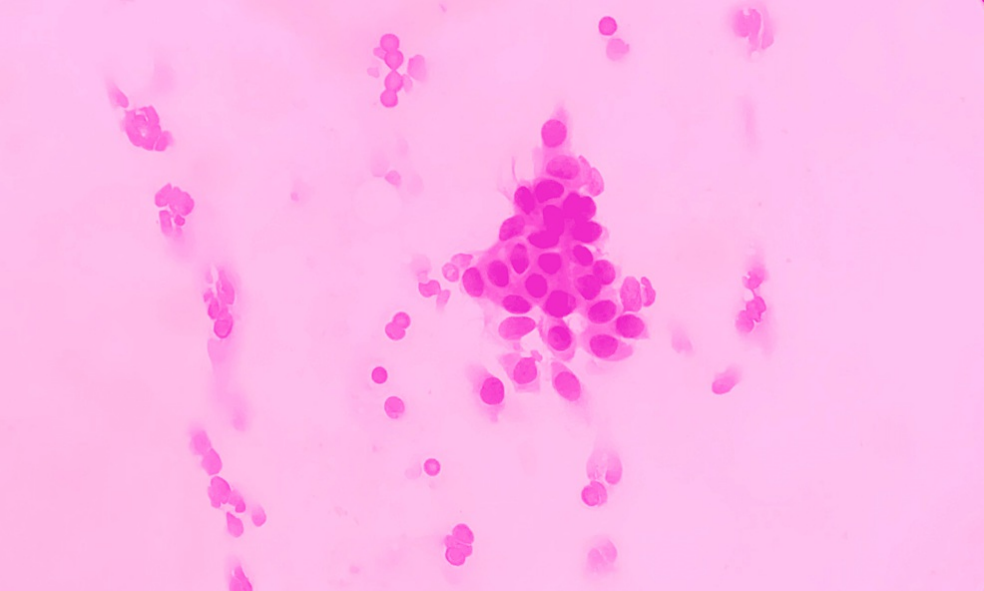
Grade I tumour – cellular features Grade I tumour with score 1 for cell size, cell uniformity, nucleoli, nuclear margin, and chromatin (FNAC - H&E x 400) FNAC: fine needle aspiration cytology; H&E: hematoxylin and eosin

**Figure 3:**
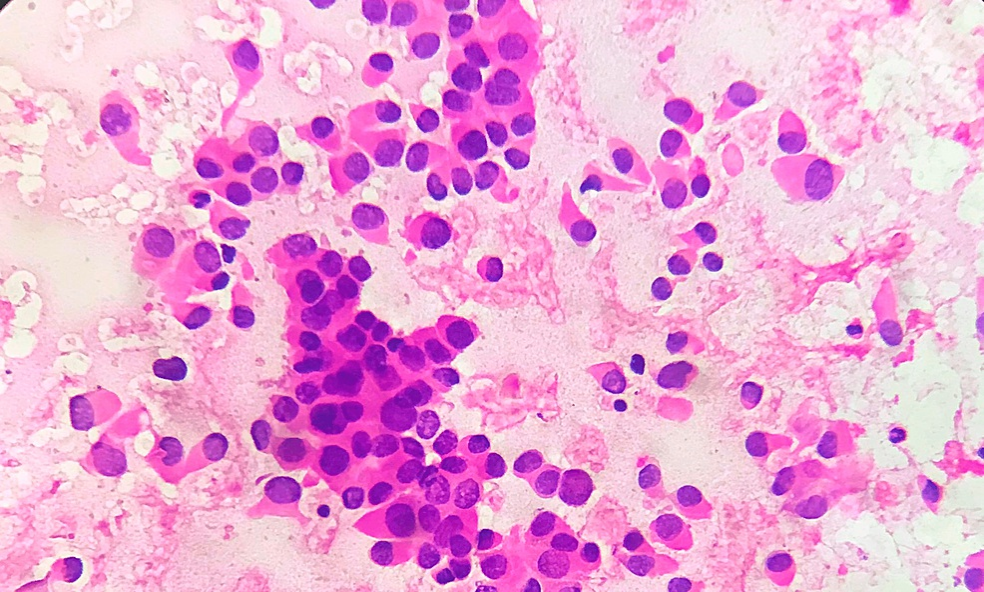
Grade II tumour cytology FNAC smear from a grade II tumour showing cells with score 2 for cell size, cell uniformity, nuclear membrane, chromatin, and cell dissociation. (FNAC - H&E x 400) FNAC: fine needle aspiration cytology; H&E: hematoxylin and eosin

**Figure 4:**
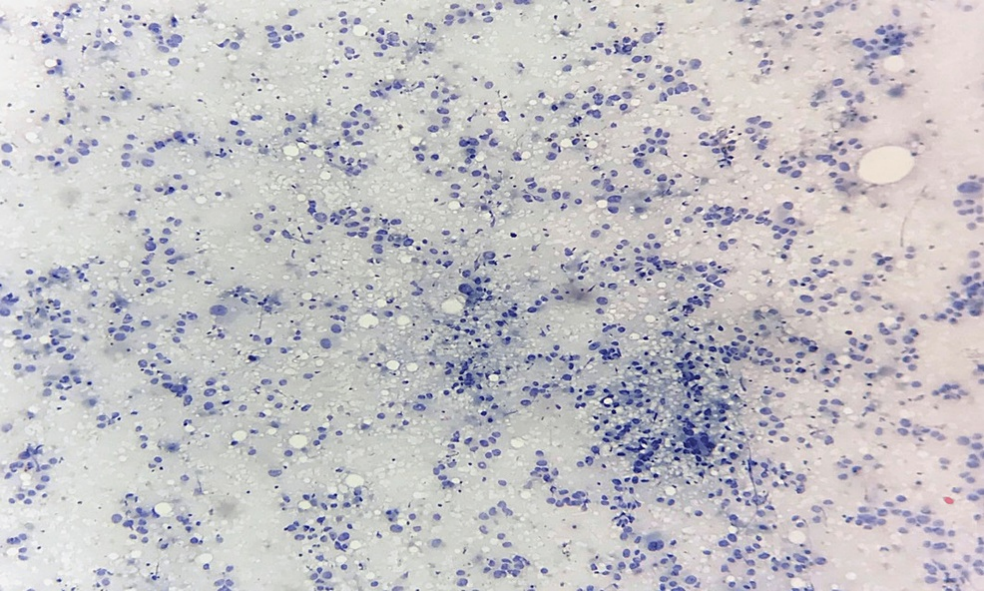
Grade III tumour – cell arrangement FNAC smear showing high cellularity with cells arranged mainly as single cells - cell dissociation score 3 (FNAC - Papanicolaou stain x 40) FNAC: fine needle aspiration cytology

**Figure 5:**
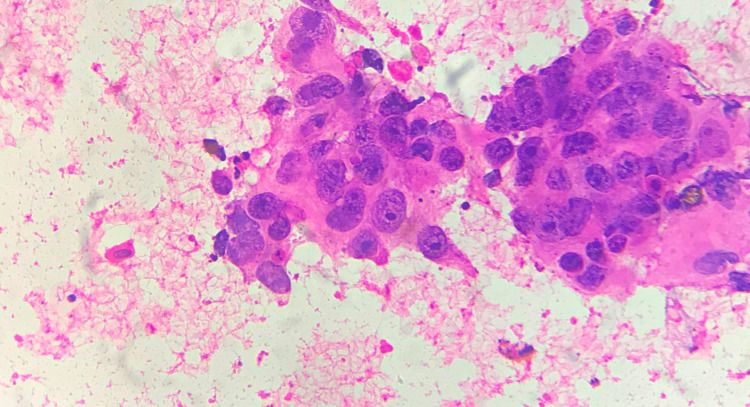
Grade III tumour – cellular features Grade III tumour with score 3 for nucleoli, cell uniformity, and cell size (FNAC - H&E x 400) FNAC: fine needle aspiration cytology; H&E: hematoxylin and eosin

**Figure 6:**
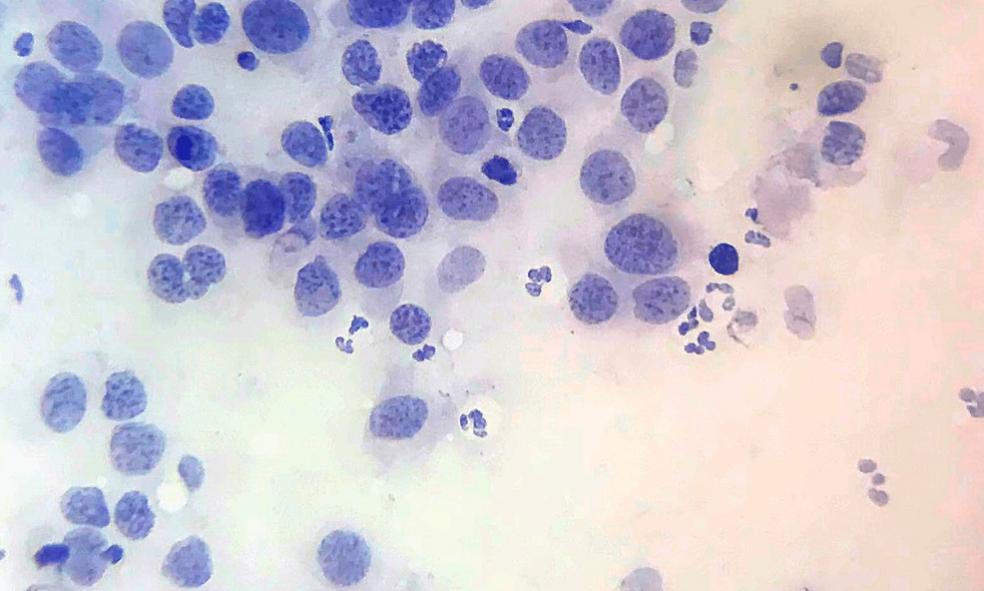
Grade III tumour – chromatin score Grade III tumour with score 3 for chromatin score (FNAC - Papanicolaou stain x 400) FNAC: fine needle aspiration cytology

Sixty-four cases of invasive breast carcinomas were identified during the study period in which both MRM and FNAC had been done. Among them, four had undergone chemotherapy before MRM and were excluded from our study. All 60 patients were females. The age of these patients ranged from 35 to 82 years with a mean age of 54.3 years. Grade II was the most common histological grade in our study (27 cases, 45%) followed by grade III (19 cases, 31.7%) and grade I (14 cases, 23.3%). Robinson’s cytological grading was done in these cases (Figures 1, 2, 3, 4, 5, 6). Grade II was the most common in Robinson’s cytological grading method as well (24 cases, 40%) followed by grade III (18 cases, 30%) and grade I (18 cases, 30%).

A comparison between histological grading and cytological grading in these 60 cases is shown in Table 3. Robinson’s cytological grade was in absolute concordance with the histological grade in 49 cases (81.7%). Even in all the 11 discordant cases, cytological grading was only one grade higher or lower when compared to the histological grade. Robinson’s cytological grading and NGS histological grading had a significant association in the Chi-square test with a p-value of 0.0001. The two grading systems showed a strong and positive correlation with Spearman's rank correlation coefficient of 0.848 with a p-value of 0.0001. The two methods also showed substantial agreement with a kappa value of 0.72. In multiple linear regression analysis, all the cytological parameters except the nuclear margin score were significantly influential in predicting the final cytological grade. The most influential parameters were nucleoli score and chromatin score with a p-value of 0.0001 (Table 4).

**Table 3:** Comparison of histological grade with cytological grade

Cytological grade	Histological grade			
	Grade I (n=14)	Grade II (n=27)	Grade III (n=19)	Total
Grade I	13 (92.9%)	5 (18.5%)	0	18
Grade II	1 (7.1%)	20 (74.1%)	3 (15.8%)	24
Grade III	0	2 (7.4%)	16 (84.2%)	18

**Table 4:** Multiple linear regression analysis of cytological parameters influencing cytology grade

Parameter	Coefficient	t-value	p-value
Dissociation score	0.169	3.466	0.001
Cell size score	0.146	2.617	0.012
Uniformity score	0.189	3.222	0.002
Nucleoli score	0.309	5.532	0.0001
Nuclear margin score	0.071	1.294	0.201
Chromatin score	0.359	5.428	0.0001
Constant	-0.931	-6.143	0.0001

When the lymph nodes from the MRM specimen were examined for metastasis, 32 cases (53.3%) showed metastasis. Of the cytological grade III tumours, 94.4% developed lymph node metastasis whereas a much lower number of cytological grade I & II tumours developed lymph node metastasis (Table 5). This difference was found to be statistically significant in the Chi-square test with a p-value of 0.0003. When we studied the association between individual cytological parameters and lymph node metastasis by Chi-square test, we found that cell dissociation score (p-0.0001), nucleoli score (p-0.001) and chromatin score (p-0.04) were the cytological parameters that showed statistically significant association.

**Table 5:** Association between cytological grade and lymph node metastasis * df- degree of freedom

Cytological grade	Lymph node status			p (df^*^=2)
	Negative	Positive	Total	
Grade I	12 (66.7%)	6 (33.3%)	18 (100%)	0.0003
Grade II	15 (62.5%)	9 (37.5%)	24 (100%)	
Grade III	1 (5.6%)	17 (94.4%)	18 (100%)	

## Discussion

The role of cytology is not just limited to diagnosis anymore. Significant prognostic information including even hormonal receptor expression in breast carcinomas can be assessed from FNAC smears [17]. If the grade of the tumour too can be obtained from FNAC smears, it will be immensely useful to the treating clinician. It will act as a complement to CNB grade whenever it is necessary to know the grade of the breast carcinoma before the excision of the tumour. Recent studies have shown a high concordance between histological grading and Robinson’s cytological grading ranging from 78% to 88% [13-16]. Our study also has shown a high concordance of 81.7% reiterating the validity of this method. This in turn indirectly indicates that Robinson’s cytological grade could be equal to or better than the CNB grade in predicting the actual histological grade of the tumour.

Histological grade I tumours had the highest concordance rate with Robinson’s cytological grading (92.9%) followed by grade III tumours (82.4%) and grade II tumours (74.1%). In all the 11 cases in which the histological and cytological grades differed; the cytological grade differed by only one grade from the histological grade. None of the cases showed more than one grade difference. Thus, none of the low-grade (grade I) tumours was graded as high-grade (grade III) or vice versa. This has been noted earlier in studies by Handa et al. and Pal et al. as well [14,15]. This ability to distinguish low-grade tumours from high-grade tumours will be of clinical significance since low-grade tumours are often resistant to chemotherapy whereas high-grade tumours respond well [4,5].

Concordance between the two grading systems in different grades of tumours

CNB often tends to underestimate grade III tumours. O’shea et al. showed in their study that 52.8% of grade III tumours were under-graded as grade II in CNB [10]. Most of the grade III tumours (84.2%) were identified correctly as grade III in Robinson's cytological grading in our study. Studies by Handa et al. and Phukan et al. had also shown that most grade III tumours were identified correctly by cytological grading [14,18]. Thus cytological grading can identify the grade III tumours that were missed by CNB and can be used as an adjunct to CNB. This will help such patients to undergo chemotherapy who otherwise might not have received it.

Among the histological grade I cases, most cases (92.9%) were identified correctly as grade I in cytology in our study. Studies done earlier have shown similar findings too [15,18]. Thus, Robinson’s cytological grading can help in excluding patients with low-grade tumours from neoadjuvant chemotherapy and prevent unnecessary adverse effects. This will be useful particularly when CNB has not been done in the patient, as may be the case in developing and resource-poor countries where only either FNAC or CNB is usually done.

In our study, the lowest concordance between cytological and histological grading was noted for the grade II tumours (74.1%), which was still in the acceptable range. Phukan et al. and Pal et al. reported a concordance of 71.4% and 82.1%, respectively, for the grade II tumours in their studies [15,18]. Although grade II is an intermediate category, the category into which some of these tumours are wrongly placed like grade I or grade III group can influence the decision on chemotherapy. Rakha et al. observed that even in histological grading, grade II tumours were frequently graded as grade I or III [5]. They explained that while scoring a biological variable, difficulties should be expected in categorizing the overlapping regions. As grade II falls in the intermediate category, difficulties are expected in grading them.

Possible reasons for the discordance noted in a few cases

Among the 11 cases that showed discordance between the two methods, eight cases were under-graded and three were over-graded in Robinson’s cytological method. Five grade II cases and three grade III cases had been under-graded. When these slides were reexamined, it was noted that in most of the cases that were under-graded, high pleomorphism score (score 3) was seen in histology but the cells showed less atypical features in the cytology smears. Probably the less atypical areas were sampled inadvertently during the FNAC procedure in these cases. Ensuring multidirectional needle passes during the FNAC procedure might help in further improving the accuracy. Such sampling issues can be more common in CNB due to the inherent nature of the procedure. This could also possibly be one of the reasons for the less concordance noted in CNB grading. Such underscoring can happen for cell dissociation score also if the more cohesive areas alone are sampled in FNAC. We could not conclude the reasons for the over-grading noted in three cases. Sampling the least cohesive areas alone or applying more pressure during smear preparation can result in a high cell dissociation score resulting in over-grading of some of the tumours.

Robinson’s cytological grading and NGS histological grading are two very different methods for breast carcinoma grading where the parameters used for grade assessment are different. Hence, achieving 100% concordance between the two entirely different grading systems might not be feasible. But still, a high concordance rate can be achieved with Robinson’s method as found in our study. Extensive multi-directional sampling during the FNAC procedure, applying optimal pressure during cytological smear preparation, immediate alcohol fixation of the cytological smears, careful attention to the microscopic findings, and strict adherence to the grading criteria will help in improving the accuracy of Robinson’s cytological grading.

Parameters influencing the cytological grade of the tumour

In multiple regression analysis, all parameters except the nuclear margin score were significantly influential in determining the final cytological grade in our study. The nucleoli score and chromatin score were the most influential among them. These two were found to be among the most influential factors determining the cytological grade in the study by Khan et al. as well along with cell dissociation score [19].

Association between cytological grade and lymph node metastasis

Lymph node metastasis is one of the major prognostic factors in breast carcinomas [20,21]. Hence, we indirectly studied the prognostic significance of Robinson’s cytological grading by studying its association with nodal metastasis. An increase in cytological grade was strongly associated (p-value of 0.0003) with the increased percentage of lymph node metastasis in our study. A similar strong association of cytological grade with lymph node metastasis has been noted in the previous studies by Robles-Frias et al. and Khan et al. [19,22]. Only a few studies are available studying the relationship between individual cytological parameters and lymph node metastasis. Robles-Frias et al. and Lingegowda et al. found a significant association between cell dissociation, cell uniformity, and nuclear margin with lymph node metastasis in their studies [22,23]. The strongest association with lymph node metastasis was for the cell dissociation score in our study (p-value of 0.0001). E-cadherin expression in the tumour cells has been shown to have a significant relationship with the cell dissociation score in cytology smears [24]. Alteration in E-cadherin expression is believed to affect the cohesiveness of the tumour cells resulting in cell dissociation. Loss of cell cohesion facilitates the metastatic cascade as the tumour cells can easily dissociate and enter the lymphatic vessels.

Our study had a few limitations. Our results were based on the analysis of 60 cases of invasive breast carcinomas. The number of cases in our study was comparable to most of the previous studies on Robinson’s cytological grading. Still, we would have liked to study more cases to add more value to our results. There are many large-scale studies on histological grading since it is used routinely. Such large-scale studies on cytological grading will be possible in future if cytological grading is done as a part of routine reporting. Another limitation of our study was that we were not able to compare the cytological grade with the histological grade. To our knowledge, there is no such study in the literature comparing the cytological grade with CNB grade and our study would have been the first of its kind. But we were not able to do it as all three types of samples were not available together in many of our cases.

## Conclusions

Histological grade and lymph node metastasis are two very important prognostic factors in breast carcinoma. Our study results showed that Robinson’s cytological grading showed strong relationship with both these prognostic factors indicating the prognostic significance of this grading system. Performing this simple cytological grading will add more prognostic value to FNAC in breast carcinomas. In places where immunohistochemistry has been standardized, hormonal receptor status can also be studied from FNAC smears along with cytological grading, thus enabling FNAC to be a major prognostic tool. More large-scale studies in the future along with long-term follow-up will help in confirming the impact of cytological grade on overall survival and disease-free survival in breast carcinoma patients.
